# Addressing myocardial infarction in South-Asian populations: risk factors and machine learning approaches

**DOI:** 10.1038/s44325-024-00040-8

**Published:** 2025-02-03

**Authors:** Rick Rejeleene, Vignesh Chidambaram, Meena Chatrathi, Amudha Kumar, Emily Lu, Ryan Pohlkamp, Mukunthan Murthi, Nitesh Gautam, Subhi Al’Aref, Xiaowei Xu, Jawahar L. Mehta

**Affiliations:** 1 Department of Information Science, University of Arkansas, Little Rock, AR USA; 2 Department of Cardiology, John H. Stroger Hospital of Cook County, Chicago, IL USA; 3 Department of Medicine, University of Arkansas for Medical Sciences, Little Rock, AR USA; 4 Division of Cardiology, Department of Medicine, Loyola University Medical Center, Maywood, IL USA; 5 Department of Medicine, Johns Hopkins School of Medicine, Baltimore, MD USA; 6 Division of Cardiovascular Medicine, Department of Medicine, University of Arkansas for Medical Sciences, Little Rock, AR USA; 7 Division of Cardiovascular Medicine, Central Arkansas Veterans Healthcare System, Little Rock, AR USA

**Keywords:** Cardiology, Acute coronary syndromes

## Abstract

Cardiovascular diseases, especially myocardial infarction (MI), are an important and up-trending public health challenge in the South Asian population. With urbanization and economic development, there has been a rise in obesity, dyslipidemia, diabetes mellitus, and hypertension in these regions, which, combined with genetic predisposition, create a unique cardiovascular risk profile among South Asians. Traditional risk assessment tools often underestimate the cardiovascular risk in South Asians due to a lack of phenotypic representation in their development. In this review, we explore the risk factors for MI in South Asians and highlight the potential role of machine learning (ML) and deep learning (DL) in enhancing diagnostic and predictive accuracy. These ML algorithms, including convolutional neural networks (CNNs) and transformer-based models, show potential in analyzing complex information from clinical characteristics, electrocardiograms (ECG), and cardiac biomarkers while integrating multimodal data. We also explore the challenges in accessing high-quality datasets and enabling applicability in clinical settings. We believe that future research should focus on developing comprehensive cardiovascular risk scores that incorporate South Asian-specific risk factors and leverage advanced ML models to enhance risk prediction, diagnosis, and management.

## Introduction

Myocardial infarction (MI) is a leading cause of death globally, contributing to nearly 17.9 million annual deaths, significantly impacting healthcare systems and economic productivity^1^. Certain ethnic populations^2,3^, particularly South Asians, experience higher rates of MI, with onset about a decade earlier, and face more severe post-MI complications compared to Western populations^4–7^. This increased risk is largely attributed to genetic predispositions, lifestyle, environmental factors, and higher rates of diabetes, dyslipidemia, and central obesity^6–8^. The INTERHEART study, which analyzed risk factors for acute MI in 52 countries, identified smoking, psychosocial stress, physical inactivity, and poor diet as key risk factors for acute MI in South Asians^6^. Despite their disproportionately higher rates of MI and post-MI complications, research focusing on the South Asian populations is relatively scarce.

Traditional risk scores, such as the ASCVD risk estimator, Framingham Risk Score, and QRISK, often underestimate cardiovascular risk in South Asians^9,10^, while the INTERHEART^6^ Modifiable Risk Score is based on case-control data^11^. Additionally, a cohort study from Norway found that the NORRISK 2 model underestimated the CVD risk in South Asian immigrants by a twofold difference^12^. Furthermore, WHO cardiovascular risk charts have been shown to misclassify high-risk South Asians as low-risk with higher prevalence^13^. These findings highlight the need for more accurate tools tailored to the unique risk profile of South Asian patients.

Given the limitations of existing risk assessment tools for South Asians, clinicians often rely on other methods and parameters. The Mediators of Atherosclerosis in South Asians Living in America (MASALA) study found the coronary artery calcium score to be a very specific marker for subclinical atherosclerosis^14^. Additionally, the INTERHEART study found the ApoB/ApoA-1 ratio to be a reliable biomarker for predicting CVD risk^15^. Thus, it is crucial to identify South Asians at risk of MI and enhance the detection of MI among symptomatic patients using advanced methods. Machine learning (ML) has shown potential in improving diagnostic and predictive capabilities. However, a significant research gap continues to exist in ML applications for CV diagnostics tailored to South Asians. This review aims to explore the risk factors for MI in the South Asian population and understand the applications of artificial intelligence (AI) and its subsets of ML and deep learning (DL) in such high-risk populations.

## Cardiovascular diseases and MI among South Asians

Cardiovascular disease (CVD) is an important cause of death among South Asians, particularly in India^16^, where 52% of deaths before age 70 are attributable to CVD, compared to 23% in Western populations^17,18^. South Asians face a 40% higher risk of mortality from MI compared to other populations^4,19,20^. The economic impact is substantial, with India projected to lose $237 billion in productivity due to CVD^4,17^.

The higher CVD burden among South Asians suggests that ethnicity by itself may be a significant risk factor^21,22^. South Asians tend to develop metabolic abnormalities at a lower BMI and waist circumference than their White counterparts^23^, suggesting genetic and ecological influences on cardiovascular outcomes. Cohort studies in rural South India identify tobacco use, alcohol consumption, hypertension, diabetes mellitus, and obesity as significant risk factors^9,20^. Low physical activity, reduced muscle mass, and central accumulation of ectopic fat also correlate with elevated risk in South Asians. While primary prevention measures, such as exercise and dietary changes, have been effective in the United States, they are underutilized in India, where evidence-based treatments are alarmingly low^17,24^.

Traditional cardiovascular risk factors, such as blood pressure, cholesterol, smoking, and diabetes mellitus, are commonly used to calculate risk scores for premature MI across all ethnicities. The Framingham risk score^25^, introduced in 1998, estimates the 10-year cardiovascular risk of individuals based on data from the Framingham heart study, a multigenerational study identifying common factors for CVD. However, this score has limitations; it underestimates CVD risk among certain populations, including South Asians^26^. However, risk factors more prevalent among South Asians, such as insulin resistance and differential body fat distribution, are often underrecognized^27^. The MASALA study highlighted that the typical South Asian vegetarian diet correlates with higher insulin resistance and lower HDL cholesterol levels, leading to a greater risk of MI^14^. The study also showed that the prevalence of CVD risk factors may differ by South Asian subgroup: hypertension was most prevalent in North Indians compared to South Indians and Pakistanis, while dyslipidemia was highest in South Indians and Pakistanis^28^. Other studies have shown similar instances of cardiovascular risk heterogeneity within the South Asian population: Population-based analysis of five major Asian groups showed that Bangladeshis have a greater prevalence of diabetes as compared to Pakistanis and Indians, while a cohort study indicated an elevated proportional hazard of ASCVD incidence compared to Pakistani and Indian subgroups^4,29^.

**Fig. 1:**
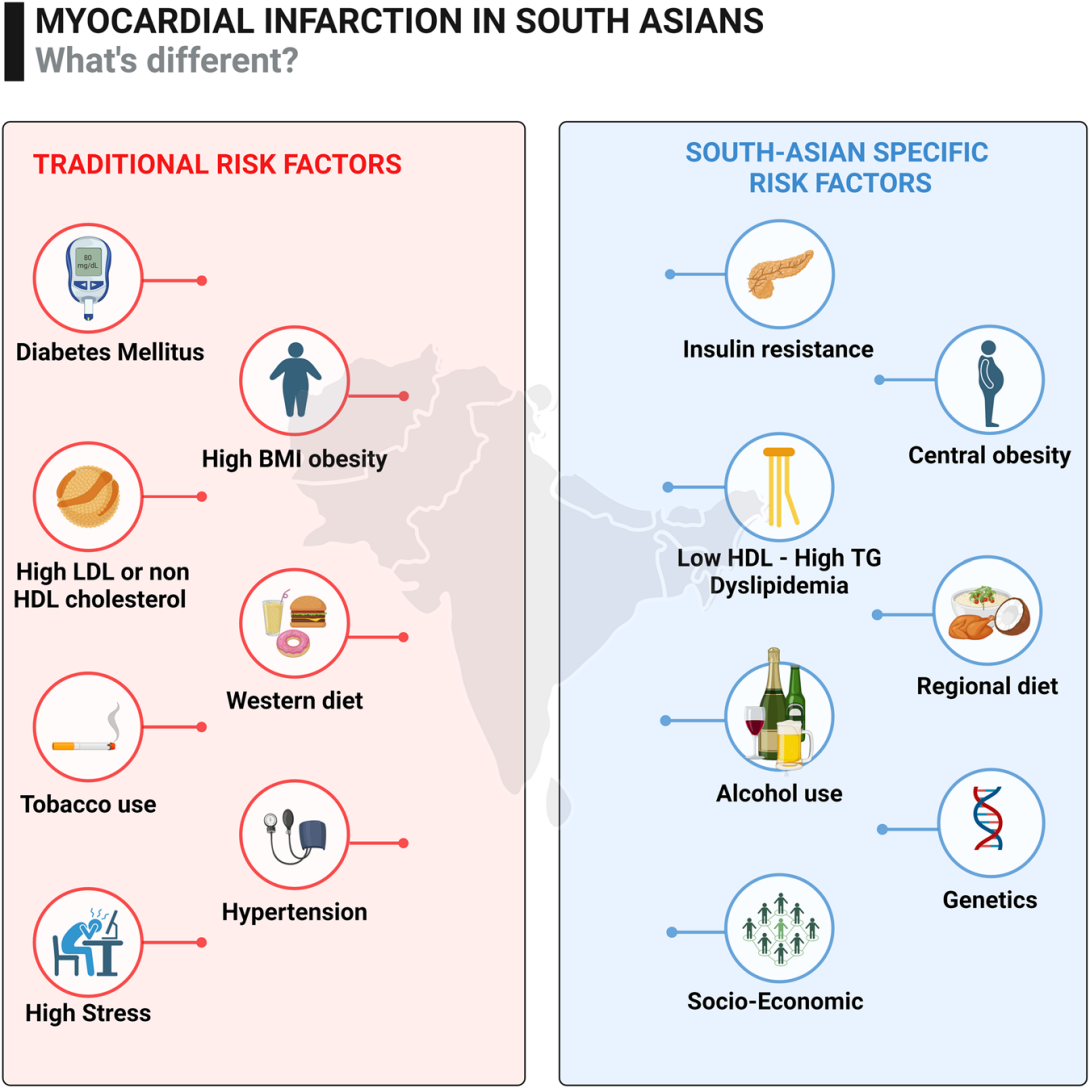
Important Traditional and South Asian specific risk factors for myocardial infarction. The left panel displays traditional risk factors for ASCVD, while the right panel highlights the risk factors that are more specific and prevalent in the South Asian population.

Developing a CVD risk calculator tailored to South Asians is crucial for addressing these unique risk factors (Fig. 1) and enhancing prevention strategies. However, research is limited by inadequate data, unreliable clinical event reporting, and insufficient information on risk factor exposure patterns, including diet, physical activity, abdominal obesity, alcohol intake, and psychosocial factors^30^. Thus, a comprehensive framework outlining the multifactorial—genetic, economic, sociological, and dietary—causes of premature CVD and MI in South Asians is needed.

## Risk factors for CVD among South Asians

### Genetic factors

The heritability of CVD risk factors is well documented among close relatives, but recent studies have focused on the genetic contribution to population-level CVD risk^31–38^. In South Asian populations, genetic variations manifest in various potential phenotypic traits such as increased visceral obesity, high waist-to-hip ratios, dyslipidemia (high triglyceride and low HDL levels), hyperglycemia, elevated blood pressure, and smaller diameter of coronary vessels^39–45^.

Genome-wide association studies (GWAS) facilitated by next-generation sequencing have identified genetic loci and polymorphisms linked to common CVD risk factors^33,34,37,38,46^. Although the exact mechanisms driving CVD risk in South Asians are not fully understood, population-specific genetic polymorphisms are believed to play a significant role^35–37^. South Asians demonstrate a higher prevalence of prediabetes and diabetes mellitus, which are significant cardiovascular risk factors. The combination of these genetic traits substantially increases the incidence of CVD in this population^16^. Comprehensive genetic research is crucial to developing targeted prevention and treatment strategies for CVD in South Asians.

Research indicates that common disease variants account for only a modest portion of total disease heritability. There is substantial interest in the impact of rare disease variants, which are more likely to differ between ethnic groups^47–54^. Further genetic analysis of the South Asian population is essential to elucidate the extent to which these polymorphisms contribute to CVD risk^55^. Additionally, the South Asian population is genetically diverse and not homogenous, necessitating detailed subgroup analyses^19^.

### Socioeconomic factors

Over the past fifty years, South Asia has transitioned from an agrarian-based economy to one dominated by manufacturing and service sectors, leading to significant reductions in physical activity and changes in dietary habits^56,57^. This economic shift has been linked to an increase in MI among the South Asian population^57,58^. Despite economic advancements, South Asia remains a significant center of global poverty, with 389 million people classified as multidimensionally poor (MPI poor) as of 2023, accounting for over 35% of the global MPI poor population^56,59^. Low socioeconomic status (SES) in the region is strongly associated with increased CVD risk factors^60^, including poor diet quality, substance abuse, reduced health screening, and mental health disorders^61–66^. Low SES during childhood is also linked to higher blood pressure in adulthood^62^. The prohibitive cost of CVD care exacerbates poverty, leading to catastrophic healthcare spending and lower employment rates in affected households. This is worsened by the fact that members of CVD-affected households also experience lower rates of employment as well^67–70^.

Consequently, more studies exploring the impact of SES on CVD risk in South Asian populations are necessary to understand deviation from global trends and how economics impact population-level CVD risk in South Asians. Interestingly, some studies in South Asia have revealed that high SES, rather than low SES, is associated with a higher risk of CVD and related risk factors, including obesity, physical inactivity, diabetes mellitus, hypertension, and dyslipidemia^61,63^.

Incorporating economic factors into cardiovascular risk scores is crucial for providing a comprehensive understanding of an individual’s risk profile, capturing the interplay between financial stability and health outcomes. This will enable more effective prevention and management strategies tailored to the unique needs of the South Asian population.

### Dietary factors

The intricate relationship between diet and MI among South Asians necessitates an in-depth examination, considering the regional diversity of dietary patterns. South Asian diets traditionally include a variety of vegetables, pulses, cereals, and potatoes rich in whole grains, fiber, and complex carbohydrates, with notable regional distinctions. However, urbanization and advancements in food processing have led to adverse lifestyle and diet changes, such as increased processed food intake and deep-frying cooking^71^. This refined carbohydrates-based diet, rich in sugar and saturated and trans fatty acids, and low in proteins, monounsaturated fatty acids (MUFAs), and ω-3 polyunsaturated fatty acids (PUFAs), is suggested to contribute to elevated CAD risk, obesity, and insulin resistance in South Asians^71,72^. As grains are a staple to the South Asian diet, comprising 60-70% of total energy intake, micronutrient-poor refined grain intake contributes to the increased cardiometabolic risk associated with elevated triglycerides, lower HDL cholesterol, and prevalence of metabolic syndrome in South Asians^73^. While Kanaya et al.^74^ and Molina et al. ^75^ did not demonstrate a direct association of HDL-to-triglyceride ratio with coronary artery calcification, the low HDL-to-triglyceride ratio was noted to be an important surrogate marker of insulin resistance in the South Asian population by Flowers et al.^76^.

Regional dietary habits play a central role in understanding MI and cardiovascular risk among South Asian populations. The Pakistan Risk of Myocardial Infarction Study (PROMIS) highlighted genetic determinants, lifestyle, and dietary factors as critical determinants of CAD in South Asians, linking high-fat diets with increased MI risk^77^. In Bangladesh, younger adults with acute MI showed significant associations with unhealthy dietary habits, including high rice and beef consumption and lower intakes of fruits and vegetables^78^. A study in Sri Lanka also indicated that high dietary intake of meat, along with alcohol consumption and smoking, were significant risk factors for MI among young males^79^. Further, in a cross-sectional analysis conducted by Daniel et al. of dietary habits across Delhi, Mumbai, and Trivandrum, regional variations in diet composition was found to have significant associations with chronic disease outcomes^80^.

Specifically, South Indian diets rich in pulses and vegetables were inversely associated with hypertension and diabetes mellitus prevalence. In contrast, diets rich in high glycemic index foods such as high-fat dairy, and fried foods, were linked to increased abdominal adiposity and a high cardiometabolic risk profile. Other studies have affirmed that high-fat intake—including high sugar intake and use of ghee rich in saturated fat—and low consumption of fruits and vegetables are risk factors for premature cardiovascular events such as MI in young South Asians^5,81^. Thus, dietary patterns among South Asian populations are complex and vary significantly by region. Low-fat, pulse-based diets in southern regions provide protective benefits against diabetes mellitus and hypertension, while high-fat diets in northern regions contribute to a higher cardiovascular risk profile. These findings emphasize the need to integrate dietary habits into the risk score to address the unique risk factors specific to different South Asian communities.

In summary, we propose developing a novel risk score specifically designed to predict the risk of MI among South Asians^82^. By incorporating major risk factors identified in prior research, including genetic, economic, sociological, and dietary factors, a risk score metric that accurately quantifies the odds of a South Asian individual experiencing an MI, can be obtained. Integrating AI will be beneficial for understanding and capturing the increased risk, as it can enhance predictive accuracy and identify complex patterns in the data. This approach requires thorough validation and depends on data quality.

## What is AI?

AI is an interdisciplinary field encompassing computer science, statistics, psychology, neuroscience, philosophy, control theory, and mathematics. It aims to perform tasks that typically require human intellect, such as problem-solving, building intelligent agents, navigating complex environments, knowledge representation, inference, planning, visual perception, decision-making, and language translation^83^.

ML, a subset of AI, involves developing algorithms and models to identify implicit patterns or relationships in data with minimal human intervention. ML consists of a model, a learning algorithm, and a task^84^. In contrast to traditional programming, ML algorithms learn from datasets and are able to explore both linear and non-linear relationships between variables. ML is categorized into supervised and unsupervised learning. Supervised learning algorithms use labeled datasets to map inputs to outputs, while unsupervised algorithms uncover the underlying structure of datasets without labeled data and is used to explore hidden relationships and patterns among data.

DL, a subset of ML, employs artificial neural networks to represent multiple levels of abstraction through computational models^85^. It can be used in both supervised and unsupervised fashions and consists of feature extraction and classification. Artificial Neural Networks (ANNs) comprise an input layer, one or more hidden layers, and an output layer, with initial training weights and tuning hyperparameters.

ANNs utilize activation functions, allowing DL to model non-linearity. DL algorithms are trained using backpropagation through gradient descent, where backpropagation computes the gradient of the loss function concerning weights and biases, and gradient descent iteratively updates these parameters to improve feature extraction and prediction accuracy.

DL has been employed for cardiovascular image interpretation and the development of models combining clinical and imaging parameters to predict cardiovascular outcomes^86^. In our review, we explore the ML algorithms, their applications in diagnostic methods for CVDs, and their possible use for more accurate and efficient MI risk prediction and diagnosis in the South Asian population.

## Diagnosis of MI

### Leveraging ML for improved MI diagnosis in South Asian populations

ML holds significant promise for enhancing MI diagnosis in South Asians, who experience higher incidence and earlier onset compared to other ethnic groups^5^. Traditional diagnostic tools often fail to account for the unique genetic, environmental, and lifestyle factors contributing to this elevated risk. ML algorithms, particularly convolutional neural networks (CNNs)^87^, can analyze complex data such as electrocardiograms (ECG) and cardiac biomarkers with high precision, identifying subtle patterns that may be missed by clinicians^88^. Table 1 provides a comprehensive overview of various ML models and techniques, along with their advantages and limitations.

**Table 1:** Overview of machine learning models and techniques with their advantages and limitations

Model/Technique	Application	Advantages	Limitations
**Basic models**			
Logistic Regression^123,124^	Risk prediction and prognosis in MI patients	Easy to interpret, widely used in clinical settings	Limited to linear relationships, less accurate for complex data
Naive Bayes^125^	Classification of MI outcomes using probabilistic models	Simple to implement, fast computation	Assumes feature independence, less effective with correlated data
Decision Trees^109^	Risk stratification and decision support for MI using decision trees	Easy to interpret, can handle both numerical and categorical data	Prone to overfitting, less accurate than ensemble methods
K-Nearest Neighbors (KNN)^126^	Patient similarity analysis and risk prediction in MI using distance metrics	Simple to implement, effective with small datasets	Computationally intensive with large datasets, sensitive to noise
**Intermediate models**			
Support Vector Machines (SVMs)^127^	MI diagnosis and arrhythmia detection using ECG signals	Robust to overfitting, effective with high-dimensional data	Requires careful tuning, not suitable for large datasets
LASSO^128^	MI diagnosis based on ECG features	Performs feature selection, reduces overfitting	Can eliminate important features, requires careful tuning
Bootstrapped Aggregation (Bagging)^129^	Improves predictive accuracy and reduces overfitting by combining multiple models	Reduces overfitting, improves accuracy	Requires large computational resources, complex to implement
Random Forest^108,130^	Feature selection and risk factor analysis in MI	Handles large datasets well, reduces overfitting through bagging	Can be computationally expensive, less interpretable than single models
Multilayer Perceptrons (MLPs)^108^	Prognosis of MI through classification of cardiac biomarkers	Capable of modeling complex relationships	Prone to overfitting, requires significant computational resources
Gradient Boosting^108,109^	Enhances predictive accuracy for MI outcomes by combining weak models	Effective for classification tasks	Prone to overfitting, requires careful tuning
Extreme Gradient Boosting (XGBoost)^131^	Enhances risk prediction and prognosis in MI patients using advanced gradient boosting methods	High performance, efficient with large datasets	Can be prone to overfitting, requires careful parameter tuning
CatBoost^125^	Handles categorical data efficiently for predicting MI outcomes	High predictive performance, handles categorical data well	Requires careful parameter tuning, computationally intensive
LightGBM^132^	Enhances MI outcome predictions by efficiently handling large datasets	High performance, efficient with large datasets	Sensitive to hyperparameters, can overfit with noisy data
**Advanced models**			
Artificial Neural Networks (ANNs)^123,124^	Basic diagnosis and prediction tasks	High accuracy, adaptable to various data types	Requires significant computational power, risk of overfitting
Convolutional Neural Networks (CNNs)^133,134^	Analyzing and classifying ECG signals	High accuracy, can detect subtle patterns	Requires large datasets, risk of overfitting
Recurrent Neural Networks (RNNs)^135^	Processing and analyzing sequential ECG data	Good for sequential data, captures temporal dependencies	Difficulty in training due to vanishing gradient problem
Long Short-Term Memory (LSTM) Networks^136^	Advanced time-series analysis of ECG data	Captures long-term dependencies, suitable for sequential data	Computationally intensive, requires significant training data
Autoencoders^137^	Dimensionality reduction and feature extraction in ECG data	Reduces noise, improves data representation	Risk of losing important information, requires careful tuning
Stacked Autoencoders^138^	Denoising and robust feature learning in ECG signals	Improves feature robustness, handles noisy data well	Requires large training data, can be computationally expensive
Deep Belief Networks (DBNs)^134^	Unsupervised learning for ECG classification and pattern recognition	Effective in unsupervised learning, robust feature extraction	Computationally intensive, complex training process
Transformer-based Models^122^	Integration of multi-modal data and natural language processing tasks	Handles sequential data well, contextual understanding	Computationally expensive, complex architecture
Generative Pre-trained Transformers (GPT-4)^122,139^	Advanced medical natural language processing tasks	High accuracy in text generation and comprehension tasks	Requires extensive training data, potential ethical considerations

*ANNs* Artificial Neural Networks, *CatBoost* Categorical Boosting, *CNNs* Convolutional Neural Networks, *DBNs* Deep Belief Networks, *ECG* Electrocardiogram, *GPT-4* Generative Pre-trained Transformer 4, *LightGBM* Light Gradient Boosting Machine, *LSTM* Long Short-Term Memory, *MI* Myocardial Infarction, *MLPs* Multilayer Perceptrons, *RNNs* Recurrent Neural Networks, *XGBoost* Extreme Gradient Boosting.

MI diagnosis traditionally relies on clinical history, ECG changes, and biomarkers like troponins^89^. However, these methods can yield inconclusive results, particularly in atypical cases. Gupta et al. noted that South Asian patients with MI may take longer to seek medical attention after the onset of symptoms, which can complicate timely diagnosis and management^90^. ML models can enhance diagnostic accuracy by integrating multimodal data from ECG, and biomarkers, providing a comprehensive assessment in a shorter time frame^91^. ML algorithms have shown promise in diagnosing myocardial infarction (MI) and predicting short-term outcomes, demonstrating significant improvements over traditional methods, as detailed in Tables 2 and 3. These advancements highlight the potential for tailored approaches to enhance cardiovascular care and management in diverse patient populations. This integrative approach could be crucial for the South Asian population, where ML can improve the identification of atypical presentations^90^.

**Table 2:** Studies assessing machine learning algorithms for acute myocardial infarction diagnosis.

Study	Source of Patient Population	Study Population	Study Design	Machine Learning Model Used	Outcome Measured And Comparison made	Main results
**Björkelund et al**.^124^	Hospital records from emergency departments in Sweden (*N* = 5695)	Patients with chest pain in the emergency department	Retrospective cohort	ANN, and logistic regression model using Python 3.7	Diagnosis of NSTEMI (Compared ML algorithms with European guideline recommended 1 and 3-hour hs-cTnT levels)	ANN and logistic regression had similar AUCs. Compared to recommended algorithms, ANN led to a 9.2% relative decrease in the intermediate risk group, while logistic regression did not substantially decrease the intermediate group size.
**Capretz et al**.^123^	Hospital records and regional patient records from Europe (*N* = 9519)	Patients with chest pain in the emergency department	Retrospective cohort	CNN, ANN, and logistic regression	Prediction of AMI or death within 30 days (Compared ML algorithms with ESC 0h arm for hs-cTnT levels)	ESC 0h (< 5 ng/L) identified 1123 (47.2%) patients for rule-out, with a 98.9% sensitivity and 99.8% NPV for AMI or death within 30 days. ANN had an AUROC of 91.9, ruling out 1109 (46.6%) patients. CNN had an AUROC of 93.9, ruling out 1309 (55.0%) patients. CNN, based on age, sex, ECG, and first blood tests (hs-cTnT, glucose, creatinine, hemoglobin), identified 60% of patients for safe and early rule-in or rule-out of 30-day AMI or death.
**Choi et al**.^140^	EMS cardiovascular registry and hospital emergency department records from South Korea	Patients with suspected acute MI	Retrospective cohort	MLP (Multilayer Preceptron), XGB (Extreme gradient boosting), logistic regression	Diagnosis of AMI at time of ED discharge	XGB and MLP models had superior performance compared to logistic regression. XGB model showed the best performance in predicting AMI at the prehospital stage. A-type models had AUCs of 0.867 (XGB) and 0.863 (MLP). B-type models had AUCs of 0.837 (XGB) and 0.836 (MLP).
**Than et al**. ^108^	Data from multiple international cohorts (*N* = 11,011)	Patients with suspected acute MI	Prospective cohort	Gradient boosting algorithm	Diagnosis of AMI and comparison with ESC rule out pathways	MI^3^ model had an AUC of 0.963. MI^3^ identified low-risk patients with a sensitivity of 97.8% and NPV of 99.7%, and high-risk patients with a specificity of 96.7% and PPV of 71.8%. MI^3^ outperformed the ESC 0/3-hour pathway.
**Doudesis et al**.^109^	High-STEACS trial cohort from Scotland	Patients with suspected Acs	Prospective cohort	Gradient boosting algorithm	Diagnosis of MI during index visit and subsequent MI or cardiovascular death at 1 year (Compared MI³ algorithm with conventional diagnostic pathways)	MI³ had an AUC of 0.949. MI³ identified 62.5% of patients as low-probability for myocardial infarction with a sensitivity of 99.3% and NPV of 99.8%, and 14.3% as high-probability with a specificity of 95.0% and PPV of 70.4%. High-probability patients had significantly higher rates of subsequent myocardial infarction or cardiovascular death at 1 year.

*ANN* Artificial Neural Network, *AMI* Acute Myocardial Infarction, *AUROC* Area Under the Receiver Operating Characteristic curve, *ACS* Acute Coronary Syndrome, *CNN* Convolutional Neural Network, *ED* Emergency Department, *EMS* Emergency Medical Services, *ESC* European Society of Cardiology, *hs-cTnT* high-sensitivity Cardiac Troponin T, *MLP* Multilayer Perceptron, *MI* Myocardial Infarction, *MI³* Myocardial Infarction Prediction Model 3, *NPV* Negative Predictive Value, *NSTEMI* Non-ST-Elevation Myocardial Infarction, *PPV* Positive Predictive Value, *XGB* Extreme Gradient Boosting.

**Table 3:** Studies assessing machine learning algorithms for short-term (in-hospital) outcomes of acute myocardial infarction.

Study	Source of Patient Population	Study Population	Study Design	Machine Learning Model Used	Outcome Measured	Main results
Chen et al.^125^	Affiliated Hospital of Zunyi Medical University, China (*N* = 438)	STEMI patients with T2DM	Retrospective cohort	Random forest, CatBoost, Naive Bayes, XGBoost, GBC, Logistic regression	In-hospital mortality	CatBoost model demonstrated the best predictive performance with an AUC of 0.92, accuracy of 0.93, precision of 0.79, and F1 value of 0.57. CatBoost outperformed other models and the GRACE risk score.
Li et al.^141^	Chinese Acute Myocardial Infarction (CAMI) registry data (*N* = 18744)	STEMI patients	Retrospective cohort	Generalized linear models, decision tree models, Bayes models	In-hospital mortality	Machine learning models (GLM, decision tree, Bayes) achieved higher prediction performance (AUC = 0.80 ~ 0.85) than traditional models. Models were also easily interpretable for clinical decision support.
Zhu et al.^129^	Taizhou Hospital and Enze Hospital, China (*N* = 5836)	Patients with First acute MI	Retrospective cohort	LR, RF, XGB, SVM, MLP, GBM, Bagging model	In-hospital mortality	Bagging model had the highest AUROC (0.932) and AP (0.63). Bagging showed high sensitivity (0.881) and specificity (0.864). The top predictors were D-dimer, BNP, cardiogenic shock, neutrophil, prothrombin time, BUN, cardiac arrest, and phosphorus.
Aziz et al.^142^	National Cardiovascular Disease Database for Malaysia registry (*N* = 12,368)	STEMI patients	Retrospective cohort	Random forest, SVM, logistic regression, ANN	Prediction of in-hospital, 30-day, and 1-year mortality among STEMI patients (Compared ML models with TIMI risk score)	ML models outperformed TIMI risk score. For in-hospital, 30-day, and 1-year mortality, AUCs were 0.88, 0.90, and 0.84 respectively for ML models vs. 0.81, 0.80, and 0.76 for TIMI. Common predictors were age, heart rate, Killip class, fasting blood glucose, prior PCI, and diuretics.
Zhao et al.^143^	Tianjin Chest Hospital, China (*N* = 5708)	STEMI patients	Retrospective cohort	SVM, LR, DT, RF	Prediction of in-hospital mortality among STEMI patients (Compared models trained with full set vs. simplified set of features and with vs. without RUS)	SVM with RUS achieved the best performance in terms of G-mean (84.93%) and AUC (0.919). Models trained with RUS outperformed those without RUS in sensitivity.
Bai Z et al.^132^	Affiliated Hospital of Zunyi Medical University, China (*N* = 2282)	STEMI patients with late cardiogenic shock	Retrospective cohort	LR, LASSO, SVM, LightGBM, XGBoost	Prediction of in-hospital cardiogenic shock (CS) among STEMI patients (Compared multiple ML models)	The LASSO model showed the best predictive performance with an AUC of 0.822 and accuracy of 0.931. The LASSO nomogram demonstrated good discrimination and calibration, with a C-index of 0.811.
Kasim et al.^144^	Data from multiple international cohorts (*N* = 20,000)	STEMI patients	Prospective cohort	Random Forest, SVM, ANN	Prediction of 30-day mortality among acute MI patients (Compared ML models with traditional risk scores)	Random Forest model achieved the highest AUC (0.93) and outperformed traditional risk scores. Common predictors included age, heart rate, blood pressure, and biomarkers.
Hadanny et al.^145^	Hospital records from multiple centers in Israel (*N* = 1200)	STEMI patients	Retrospective cohort	XGB, SVM, LR	Prediction of in-hospital mortality and major adverse cardiovascular events (Compared ML models with standard clinical assessments)	XGB model had the highest predictive accuracy with an AUC of 0.87. The model showed significant improvement over standard clinical assessments.

*ANN* Artificial Neural Network, *AUROC* Area Under the Receiver Operating Characteristic Curve, *BNP* B-type Natriuretic Peptide, *BUN* Blood Urea Nitrogen, *CS* Cardiogenic Shock, *DT* Decision Tree, *ED* Emergency Department, *ESC* European Society of Cardiology, *GBM* Gradient Boosting Machine, *GLM* Generalized Linear Model, *GRACE* Global Registry of Acute Coronary Events, *hs-cTnT* High-sensitivity Cardiac Troponin T, *LASSO* Least Absolute Shrinkage and Selection Operator, *LR* Logistic Regression, *ML* Machine Learning, *MLP* Multilayer Perceptron, *NPV* Negative Predictive Value, *PCI* Percutaneous Coronary Intervention, *PPV* Positive Predictive Value, *RF* Random Forest, *RUS* Random Under-Sampling, *SVM* Support Vector Machine, *STEMI* ST-Elevation Myocardial Infarction, *T2DM* Type 2 Diabetes Mellitus, *TIMI* Thrombolysis in Myocardial Infarction, *XGB* Extreme Gradient Boosting.

Personalized diagnostic approaches enabled by ML consider individual risk factors unique to South Asians, including higher rates of diabetes and central obesity, tailoring assessments to the specific risk profile of each patient^90^. This personalization enhances the relevance and accuracy of diagnoses. Additionally, ML reduces diagnostic errors by providing consistent and objective analyses of diagnostic data, minimizing the risk of human error^92^. The complexity of integrating and interpreting multiple diagnostic tests often leads to errors; however, ML algorithms can automatically interpret ECG signals and imaging results, ensuring more reliable diagnoses.

Moreover, ML optimizes healthcare resources by prioritizing high-value diagnostic tests and reducing unnecessary procedures, which is particularly beneficial in resource-limited settings in South Asian countries^93^. This efficiency ensures patients receive appropriate and timely care. Incorporating ML into MI diagnosis also addresses health disparities in access and quality of cardiovascular care. ML-driven mobile health applications can facilitate early detection and continuous monitoring of cardiovascular health in remote or underserved areas^93^. It must be noted that Machine Learning for risk prediction has limitations when applied in clinical settings. Traditional ML models may not be able to overcome confounding factors or biases that are inherent to the training data. Careful consideration of the quality of training data is necessary, and additional strategies by the clinician must be made to mitigate these biases to ensure accurate outcomes in clinical decision-making.

In summary, ML could significantly enhance MI diagnosis in the South Asian population by improving diagnostic accuracy, integrating multimodal data, and identifying atypical presentations^94^. ML may provide personalized diagnostic approaches, reduce errors, and optimize healthcare resources. These algorithms can generate differential diagnoses, suggest high-value tests, and minimize repeated testing. Beyond diagnostics, ML may be able to predict patient outcomes and assist in clinical decision-making by providing insights based on large datasets, helping clinicians tailor treatment plans effectively^95^. These advancements are crucial for addressing the unique challenges faced by South Asians in cardiovascular health, ensuring better clinical outcomes for this high-risk population.

### Electrocardiogram

Electrocardiograms (ECG) are non-invasive, low-cost tools essential for detecting abnormal cardiac activity through analysis of electrical impulses, providing important information for MI diagnosis^89^. However, traditional ECG interpretation is limited by the complexity and variability of cardiac signals, which can obscure subtle but clinically significant patterns^88^. ML algorithms can enhance ECG analysis by segmenting and classifying signals using supervised and unsupervised learning, thereby improving diagnostic accuracy and clinician productivity^88,96^.

Supervised learning algorithms, such as logistic regression, support vector machines, ANNs, and random forests, are widely used for ECG classification, aiding disease diagnosis and risk stratification^92,94,97^. Unsupervised learning methods, including principal component analysis (PCA), help discover hidden patterns in ECG datasets by reducing dimensionality and managing large volumes of signals. This approach can identify phenotypic subtypes in conditions like hypertrophic cardiomyopathy and cluster biomarkers associated with MI^97,98^. Nonetheless, overfitting remains a challenge in ML, where models perform well on training sets but poorly on unseen data.

DL architectures, such as autoencoders, deep belief networks (DBNs), convolutional neural networks (CNNs), and recurrent neural networks (RNNs), mitigate overfitting and improve ECG classification through hierarchical feature extraction^98,99^. RNNs perform recursion by directing the sequence of nodes connected in a chain, where the output of each node is used as input for the next. RNNs are suitable for time-series data like ECG signals but face limitations in learning long-term dependencies for long sequence inputs. Long Short-Term Memory (LSTM)^100^ networks address this issue and are popular algorithms for ECG diagnosis. By combining forward and backward LSTMs, bidirectional LSTM^101^ networks analyze ECG signals using information from both the future and the past, enhancing the accuracy and robustness of ECG signal analysis.

#### Advanced DL techniques for ECG classification

Advances DL approaches have demonstrated exceptional performance in ECG classification, addressing challenges such as overfitting and the need for hand-crafted features. Stacked autoencoders, comprising multiple layers of autoencoders and a classifier, learn robust representations through complex relationships^102^. For ECG classification, denoising and sparse autoencoders can enhance feature detection^102^.

In addition to stacked auto-encoders, DBNs, composed of stacked restricted Boltzmann machines (RBMs), are generative neural networks utilizing unsupervised learning modules to predict probability distributions over inputs. During pre-training and fine-tuning of the ECG dataset for classifying MI, each RBM is trained sequentially using contrastive divergence to learn initial weights, followed by backpropagation to optimize discriminative performance with labeled data. DBNs perform well even with corrupted data, although achieving high classification accuracy can be challenging^98^.

CNNs, a form of feedforward neural network characterized by a hierarchical structure, are predominantly used for image-based datasets in DL. Unlike fully connected neural networks, where every layer is connected, CNNs learn filters that apply operations to each sub-region of the input. Architecturally, CNNs consist of convolutional filters, pooling modules, and fully connected layers. Convolutional layers perform the convolution of each sub-region of the input with a filter kernel, extracting features from the input provided by previous layers^103^. Pooling layers reduce the dimensions of feature maps, retaining essential information while lowering computational complexity. Fully connected layers integrate features extracted by convolutional and pooling layers for final classification.

Several advanced CNN architectures, like AlexNet and VGG-Net, improve performance by using deeper networks and smaller convolutional filters^103,104^. A deep CNN developed by Acharya et al.^94^ for detecting MI achieved an accuracy of 93.53% using ECG^94^. Xiong et al.^105^ reported the highest accuracy for MI localization using ResNet^106^, achieving 99.99% accuracy, followed by 99.95% using a convolutional neural network.

Despite these accomplishments, CNNs face several limitations in MI detection. Access to high-quality and large datasets is a major challenge. The performance of DL algorithms often lacks standardized measurement and comparison across different experimental settings, many of which are arbitrary. Additionally, robustness is an issue, as ECG waveforms in clinical settings can be deformed by external forces, potentially affecting DL performance in real-life environments. In summary, advanced ML and DL techniques significantly enhance ECG classification and cardiac disease diagnosis, though challenges such as data quality, model robustness, and interpretability remain.

### Cardiac biomarkers

Cardiac biomarkers are proteins released into the blood following cardiac injury, crucial for diagnosing acute MI^107^. Aydin et al. identified myoglobin, fatty acid-binding protein (FABP), and glycogen phosphorylase BB (GPBB) as early biomarkers for MI, while troponin T (TnT) and troponin I (TnI) are definitive biomarkers for MI diagnosis^107^. Troponin I (TnI) is currently regarded as the gold standard for diagnosing acute MI, with elevated troponin levels at 0, 3, and 6 hours, offering critical insights into the severity and timing of myocardial injury.

The myocardial-ischemic-injury index (MI3), an ML algorithm developed by Than et al.^108^, incorporates age, sex, and cardiac high-sensitivity troponins measured in paired samples. This model was trained on a cohort of 3,013 patients and validated on 7,998 individuals presenting with suspected MI. MI occurred in 10.6% of patients in the test set and 13.4% in the training set. The MI^3^ index effectively identified low and high-risk patients using a gradient boosting algorithm, allowing for individualized and objective assessments of the likelihood of MI, thus enhancing patient care. The MI^3^ algorithm computes a value between 1 and 100, reflecting an individual’s likelihood of an MI diagnosis^109^. Despite its potential, gradient boosting is prone to overfitting and is computationally expensive. Current diagnosis of MI relies on fixed troponin thresholds, which does not account for varying troponin levels based on age, sex, or time.

Doudesis et al.^109^ validated the MI3 algorithm through an exploratory analysis using a multicenter randomized trial conducted in Scotland. Among 20,761 patients, 3,272 (15.8%) had an MI. The MI3 algorithm demonstrated a clear area under the receiver-operating characteristic curve of 95%, identifying 12,983 (62.5%) patients as having a low probability of MI at a prespecified threshold, with a negative predictive value of 99.8%. Additionally, 2,961 patients were identified as having a high probability, with a positive predictive value of 70.4%. At one year, subsequent MI or cardiovascular deaths occurred more frequently in high-probability patients than in low-probability patients (17.6% vs. 0.5%; *p* < 0.001). These results suggest that the MI3 algorithm is a potential tool for improving the accuracy of MI diagnosis by accounting for varying troponin levels, age, gender, and time, thus allowing for more personalized and accurate risk assessments. Multilayer Perceptrons (MLPs) are another ML algorithm that can be used to classify cardiac biomarkers. MLPs comprise multiple layers of neurons, each fully connected to the next, allowing them to capture complex patterns in the data. These advanced algorithms can enable a more nuanced analysis of cardiac biomarkers.

In summary, leveraging cardiac biomarkers such as troponins with innovative ML algorithms like MI^3^ offers a more personalized and accurate approach to diagnosing and managing MI. These advancements hold promise for significantly improving clinical outcomes in patients with suspected acute MI. ML algorithms such as VGG-Net and CNNs utilize image datasets, achieving high accuracy in diagnosing MI^110^. However, there are limitations, such as a lack of access to quality datasets and challenges in evaluating dataset quality. Significantly less research has focused on assessing the information quality in datasets^111^, and high-quality datasets are essential for developing and training ML algorithms, particularly in MI detection. To address these challenges, a user-centric, ML-based information quality evaluation tool for assessing the quality of MI datasets across three dimensions—consistency, relevance, and accuracy—could prove useful. Consistency measured using edit distance and cosine similarity, relevance assessed by Jaccard similarity and distance, and accuracy evaluated using KL divergence, the Kolmogorov-Smirnov test, and Pearson correlation can enable us to quantify the dataset’s information quality.

### Transformer-based models in MI

Based on the Transformer model, the advanced ML algorithm categorizes MI risk into low, medium, and high probability levels. Transformer models have achieved advanced performance in various tasks, including machine translation, language generation, image classification, and object detection^111–113^. By providing a probabilistic classification, the Transformer model aids in patient stratification and tailored interventions.

Transformer-based models, originally introduced for neural machine translation, have revolutionized natural language processing (NLP) by significantly improving sequence translation accuracy through the attention mechanism^114^. This addresses fixed-length vector problems by allowing a joint soft search of the source sentence, thus improving translation accuracy. Encoder-decoder architectures have been the state of the art for translation tasks. However, while translating sequences, the decoder could not gain the context of previous vectors in a sequence of sentences. Due to this, translation accuracy was low.

Attention mechanisms overcame this limitation, enhancing translation performance by allowing a joint soft search of the source sentence without facing bottlenecks of a fixed vector, thereby improving translation accuracy. Transformers, introduced by Vaswani et al.^112^, are solely based on the attention mechanism, dispensing with recurrence and convolution entirely, achieving state-of-the-art results in machine translation^112^. Subsequently, Transformers have been applied to computer vision^113,115^ and speech recognition, achieving advanced performance across numerous ML tasks^111,116,117^.

Based on transformers, subsequent generations of language models were developed. Devlin et al.^118^ introduced Bidirectional Encoder Representations from Transformers (BERT), enabling models to gain a contextual understanding of sentence sequences in machine translation tasks and improving downstream tasks such as text classification, question answering, named entity recognition, and text summarization.

Additionally, Generative Pre-trained Transformers (GPT) by Radford et al.^117^ utilized only the decoder from Transformers to address natural language generation tasks. GPT-2 increased the capacity and size of parameters, further enhancing natural language generation^119^. GPT-3 achieved excellent results in many natural language processing tasks, while GPT-4 further increased parameter size and introduced multi-modality, handling both image and text inputs. Recent work by Bubeck et al.^120^ suggests the emergence of Artificial General Intelligence using a GPT-based ML model.

Despite the significant technical progress of language models in ML, their utilization in the field of cardiology is still in its early stages, with considerable gaps in research and application of language models. However, language models hold great potential for diagnosing and managing MI. An innovative vision transformer, HeartBEiT, developed by Vaid et al.^121^, demonstrates superior diagnostic performance for ECG analysis compared to traditional CNN architectures, particularly in low-data regimes. This model, pre-trained on a vast body of ECGs, enhances explainability and improves diagnosis by highlighting important regions of the ECG.

Selivanov et al.^122^ demonstrated the potential of language models in medical image captioning by combining radiological images with organized patient data from textual records, generating summaries that aid in diagnosis and treatment planning. Moreover, Bubeck et al.^120^ demonstrate the capabilities of GPT-4 in mastering medical natural language processing tasks, such as clinical question answering, note generation, and treatment plan generation. Training medical datasets using GPT-4 can significantly improve the prediction of MI. Beyond prediction, GPT-4 can identify at-risk patients and generate tailored treatment plans for those likely to suffer from acute MI, thereby enhancing patient care and outcomes.

In summary, leveraging advanced Transformer-based models alongside innovative ML algorithms like HeartBEiT and GPT-4 offers a more personalized and accurate approach to diagnosing and managing MI. These advancements hold promise for significantly improving clinical outcomes in patients suspected of acute MI.

## Clinical implications and future directions

The integration of ML and DL algorithms in the risk prediction, diagnosis, and short- and long-term prognosis of MI can have important clinical implications and offer significant improvements over traditional methods. ML algorithms, particularly CNNs and transformer models, have demonstrated superior performance in analyzing complex data from ECGs and cardiac biomarkers and can detect subtle patterns often missed by clinicians, improving diagnostic accuracy^94^. This helps prioritize resources, particularly in resource-limited settings in South Asia, ensuring timely and appropriate care or referral. ML algorithms specifically can help in triaging patients in the emergency department^108^. ML can offer clinical decision support by simultaneously and rapidly analyzing vast amounts of data, offering insights into treatment plans^122^. Thus, the integration of ML and DL holds promise for improving risk prediction, enhancing accuracy, and enabling efficiency in MI diagnosis and management, specifically for the South Asian population. Generative artificial intelligence (AI) powered tools, such as virtual co-pilots, can support clinicians by offering real-time, personalized health recommendations that account for individual genetic, lifestyle, and environmental factors. Furthermore, AI platforms can deliver culturally tailored health education, improving patient understanding and engagement. Integrating these advanced AI technologies into clinical practice has the potential to significantly improve secondary cardiovascular prevention strategies, particularly in high-risk populations.

Despite significant advances in ML and DL, several knowledge gaps remain. One of the main challenges is the lack of high-quality and standardized datasets. Many ML models are trained on datasets with poor consistency, thereby impacting their generalizability and robustness in clinical settings^105^ The current ML models in myocardial infarction do not capture the unique genetic, environmental, and lifestyle factors of South Asian populations, leading to underestimation of MI risk in these groups, thereby noting the need for risk assessments tailored to different ethnic groups^12^ More comprehensive ML models integrating multimodal data are required. Establishing global standards to improve the quality of datasets will enable proper training of machine learning models. The most important step is translating ML algorithms into actual clinical practice. Collaborative efforts are needed between researchers, clinicians, and policymakers to ensure the accessibility of ML tools to diverse populations.

## Conclusions

In conclusion, MI significantly impacts global health, particularly in South Asian populations with higher incidence and earlier onset. Traditional risk scores often fail to capture the unique genetic, environmental, and lifestyle factors contributing to this elevated risk. Advanced ML and DL techniques, such as CNNs and transformer-based models, show substantial promise in enhancing MI detection, prediction, and management by integrating multimodal data and identifying subtle patterns missed by clinicians. Developing tailored MI risk scores for South Asians, incorporating genetic, economic, sociological, and dietary factors, is essential for effective prevention and early interventions. Ensuring high-quality datasets is crucial for these ML models to be clinically applicable. Leveraging these advanced tools can significantly improve cardiovascular outcomes and quality of life for at-risk populations.

## Data Availability

No datasets were generated or analysed during the current study.
